# LISTEN: lived experiences of Long COVID: a social media analysis of mental health and supplement use

**DOI:** 10.3389/fdata.2025.1539724

**Published:** 2025-06-18

**Authors:** Sam Martin, Maya Janse Van Rensburg, Huong Thien Le, Charlie Firth, Abinaya Chandrasekar, Sigrún Eyrúnardóttir Clark, Samantha Vanderslott, Cecilia Vindrola-Padros, Norha Vera San Juan

**Affiliations:** ^1^Rapid Research, Evaluation, and Appraisal Lab (RREAL), Department of Targeted Intervention, University College London, London, United Kingdom; ^2^Oxford Vaccine Group, NIHR Oxford Biomedical Research Centre, University of Oxford, Oxford, United Kingdom

**Keywords:** Long COVID, mental health and social media, LISTEN method, Infranodus discourse analysis, dietary supplements and patient self-management

## Abstract

**Introduction:**

Long COVID, or Post-Acute Sequelae of SARS-CoV-2 infection (PASC), is a complex condition characterized by a wide range of persistent symptoms that can significantly impact an individual's quality of life and mental health. This study explores public perspectives on the mental health impact of Long COVID and the use of dietary supplements for recovery, drawing on social media content. It uniquely addresses how individuals with Long COVID discuss supplement use in the absence of public health recommendations.

**Methods:**

The study employs the LISTEN method (“Collaborative and Digital Analysis of Big Qual Data in Time Sensitive Contexts”), an interdisciplinary approach that combines human insight and digital analysis software. Social media data related to Long COVID, mental health, and supplement use were collected using the Pulsar Platform. Data were analyzed using the free-text discourse analysis tool Infranodus and collaborative qualitative analysis methods.

**Results:**

The findings reveal key themes, including the impact of Long COVID on mental health, occupational health, and the use of food supplements. Analysis of attitudes toward supplement use highlights the prevalence of negative emotions and experiences among Long COVID patients. The study also identifies the need for evidence-based recommendations and patient education regarding supplement use.

**Discussion:**

The findings contribute to a better understanding of the complex nature of Long COVID and inform the development of comprehensive, patient-centered care strategies addressing both physical and mental health needs.

## 1 Introduction

The COVID-19 pandemic has not only caused acute illness but has also led to a growing concern about the long-term effects of the SARS-CoV-2 virus, known as Long COVID or Post-Acute Sequelae of SARS-CoV-2 infection (PASC). Long COVID is characterized by a wide range of persistent symptoms that continue for weeks or months after the initial infection, even in individuals who experienced mild or asymptomatic cases (Gupta et al., 2020; Taquet et al., 2021; Ballering et al., 2022). The prevalence of Long COVID varies across studies, with estimates ranging from 1 in 8 (in a study of 273,618 COVID-19 survivors; Taquet et al., 2021) to 1 in 3 (in a study of 76,422 participants; Ballering et al., 2022) individuals affected who have had COVID-19. This condition presents a significant challenge to healthcare systems and has a substantial impact on the quality of life and economic wellbeing of affected individuals (Cutler, 2022).

One of the most significant consequences of Long COVID is its impact on mental health. Studies have shown that individuals with Long COVID are at an increased risk of developing mental health conditions, such as depression, anxiety, and post-traumatic stress disorder (PTSD; Taquet et al., 2021; Vindegaard and Benros, 2020). The persistent physical symptoms, coupled with the uncertainty surrounding the course and prognosis of the condition, can lead to significant psychological distress. Moreover, the social isolation, financial hardship, and disruption to daily life caused by Long COVID can further exacerbate mental health problems (Xiong et al., 2020). Research indicates that the levels of anxiety and depression among Long COVID patients can be as high as 50%, highlighting the need for targeted mental health interventions (Groff et al., 2021). A longitudinal study conducted in the United States, involving 273,618 COVID-19 survivors (mean age 46.3 years, 55.6% female) recruited from electronic health records, revealed that up to 40.6% of Long COVID patients experienced symptoms of depression, and 34.7% had symptoms of anxiety even 6 months after the onset of COVID-19-related symptoms (Taquet et al., 2021). These findings are consistent with calls for more robust mental health interventions to address the psychological and socioeconomic challenges associated with Long COVID (Hawke et al., 2022).

Beyond the psychological distress directly associated with experiencing chronic symptoms, emerging evidence suggests that Long COVID might have direct impacts on neurological functioning, with possible consequences for developing psychiatric disorders even after a relatively long time from recovery. Research has indicated neuroinflammatory processes in COVID-19 that may affect the central nervous system, potentially leading to neuropsychiatric manifestations that extend beyond the acute phase of illness (Ali Awan et al., 2021). Studies have also documented neurological complications and cognitive impairments that may contribute to psychiatric conditions through alterations in brain structure and function, further complicating the mental health landscape for Long COVID patients (De Berardis et al., 2022). These findings suggest that the mental health impacts of Long COVID may stem not only from psychosocial stressors but also from direct neurobiological mechanisms, highlighting the need for integrated approaches to assessment and treatment.

Due to significant variability in symptom presentation and the current lack of robust research, many patients have turned to alternative therapies, including dietary supplements, to manage their symptoms (Islam et al., 2020). The use of supplements such as vitamins, minerals, and herbal products has become increasingly common among Long COVID patients seeking relief from symptoms like fatigue, brain fog, and muscle pain (Lordan et al., 2021). However, as Chee et al. (2023) highlight in their systematic review of pharmacological interventions for Long COVID, the heterogeneity of symptoms and outcomes in current trials poses a challenge to establishing clear treatment guidelines (Chee et al., 2023). They note that most clinical studies target isolated symptoms rather than taking a comprehensive approach to Long COVID, and these studies often lack standardized diagnostic criteria and outcome measures, further complicating the development of universal treatment recommendations. Ethical frameworks, such as the WHO's MEURI framework (Monitored Emergency Use of Unregistered and Experimental Interventions), highlight the importance of implementing unproven interventions under rigorous ethical conditions, including informed consent, data monitoring, and equitable access. This type of framework is useful for addressing the uncertainties surrounding non-pharmacological therapies for Long COVID by balancing the urgent need for symptom relief with the ethical obligation to ensure patient safety and data transparency (Mastroleo et al., 2020). The use of food supplements without proper medical guidance may also lead to interactions with prescribed medications and potential adverse effects, underlining the need for evidence-based recommendations and improved patient education (Islam et al., 2020).

Recent studies have begun to explore the potential benefits and risks associated with supplement use among Long COVID patients. For example, some research suggests that certain food supplements, such as vitamin D and omega-3 fatty acids, may have anti-inflammatory properties that could help alleviate some symptoms of Long COVID (Islam et al., 2020). Chandan et al. (2023) have expanded the discourse on non-pharmacological therapies by highlighting a broader array of interventions used across post-viral syndromes, including Long COVID (Chandan et al., 2023). Their systematic review identifies approaches such as Pilates, resistance exercises, telerehabilitation, and neuromodulation,^1^ which have shown some efficacy in alleviating symptoms like fatigue, dyspnoea, and musculoskeletal pain—symptoms also prevalent in Long COVID. However, these findings are based on a limited number of studies, and none focused specifically on food supplements, underscoring the need for more targeted research to establish robust evidence on their effectiveness.

In parallel, qualitative studies have shed light on how patient communities contribute to understanding and navigating Long COVID. Callard and Perego (2021) highlighted the vital role of “patient-expert” communities in shaping the Long COVID narrative, where individuals actively share self-tracking data and research findings to advocate for tailored solutions (Callard and Perego, 2021). Similarly, Lewthwaite et al. (2023) propose a “Treatable Traits” framework, emphasizing the need for precision medicine approaches that address the heterogeneity of Long COVID symptoms by focusing on specific, clinically actionable traits. These frameworks highlight the growing recognition of patient-led contributions to healthcare and underscore the importance of integrating participatory research with clinical practices.

The complex interplay of mental health challenges, self-management strategies, and patient-led advocacy in Long COVID highlights the need for interdisciplinary approaches. To bridge these gaps, our study aims to explore public views on the challenges faced by Long COVID patients, particularly regarding mental health and the use of food supplements, through social media content analysis.

## 2 Materials and methods

**Figure 1:**
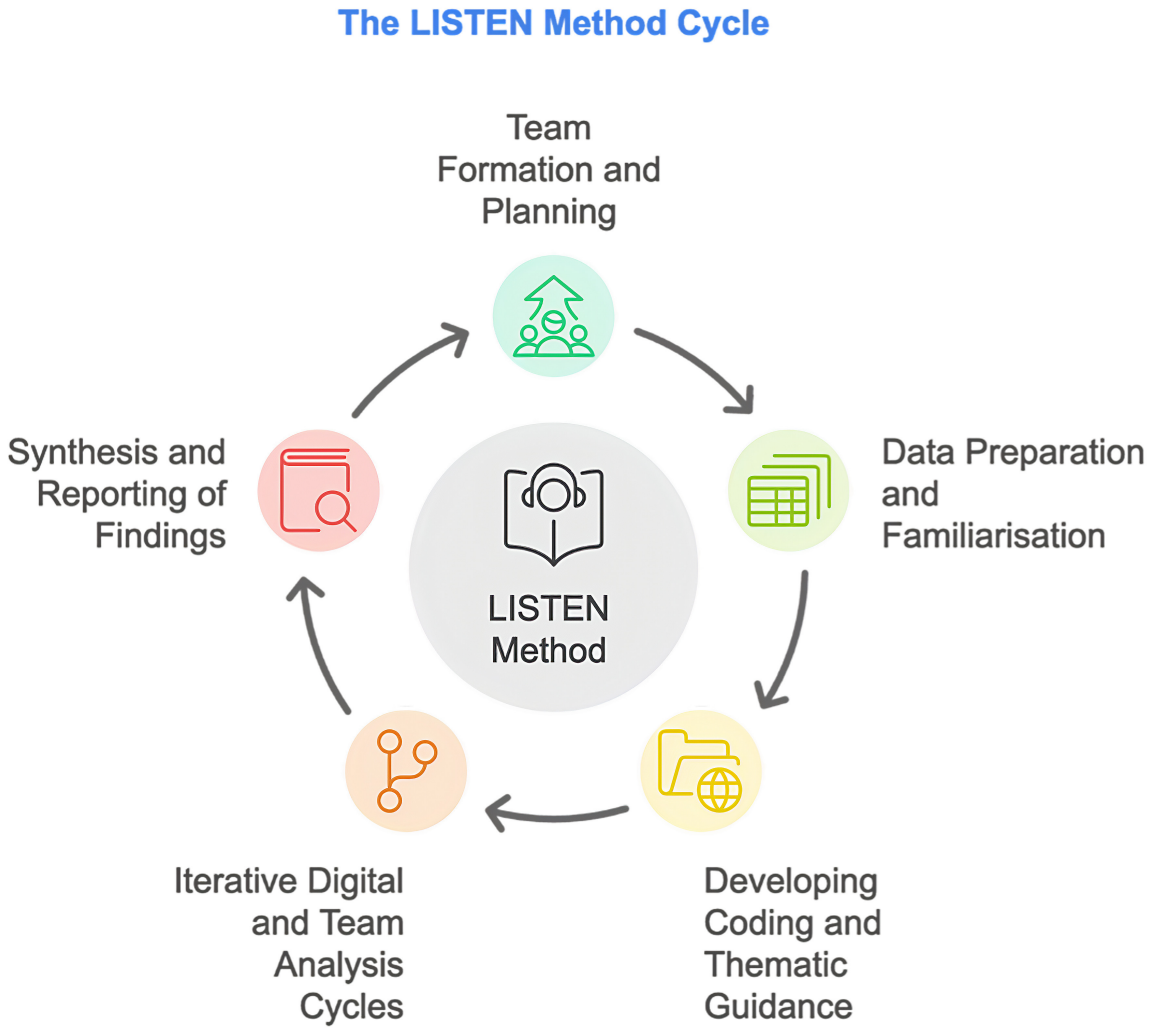
LISTEN method cycle. The LISTEN method is a five-stage qualitative research cycle designed to handle large-scale digital textual data. It combines human-led thematic analysis with digital tools like Infranodus to enhance pattern detection and theme development. “Categories” here refer to collaboratively generated codes representing key themes in qualitative data, not statistical variables or formal classifications.

Our study comprised two main components: a case study on the social media dataset and a collaborative thematic analysis. In this paper, we employed the LISTEN method [“Collaborative and Digital Analysis of Big Qual Data in Time Sensitive Contexts” (Vera San Juan et al., 2023)] to explore the landscape of Long COVID, focusing on its impact on mental health and the use of food supplements (Clark et al., 2022). The LISTEN method is an iterative qualitative approach that integrates manual and digital analysis techniques to manage large-scale qualitative datasets effectively, especially in time-sensitive contexts. The process is divided into five main stages: team formation and planning, data collection, preparation, and familiarization, development of coding and thematic guidance, iterative digital and team analysis cycles, and synthesis and reporting of findings (Figure 1). Each phase is grounded in participatory qualitative research practices, with team consensus shaping category definitions. The term “categories” in this workflow refers to interpretive thematic codes, not variables subjected to statistical modeling.

### 2.1 Team formation and planning

This stage involved assembling a multidisciplinary team, essential for ensuring a comprehensive analysis of Long COVID discourse across social media. The team included a lead qualitative researcher to oversee methodological rigor, three research assistants (RAs) who contributed to data management and initial analysis, and a digital analyst responsible for managing and interpreting social listening data from platforms like Pulsar and Infranodus.

The research assistants received training on using these digital tools, equipping them with the technical skills necessary for effective data analysis. Notably, some team members brought lived experience perspectives of mental health challenges or long-term conditions, which provided valuable context for understanding and interpreting the data. This combination of technical training, methodological expertise, and personal insight allowed the team to address the diverse and nuanced aspects of Long COVID, adapting the approach as needed to meet the specific demands of this dataset.

### 2.2 Data collection, preparation, and familiarization

Data from the Pulsar Platform was divided among team members for preliminary review and familiarization. Social media posts were filtered using inclusion and exclusion criteria (Appendix 2), focusing on content related to Long COVID, mental health, and supplement use over a 93-day period from August 1 to November 2, 2023. This period aligns with observed seasonal trends in respiratory virus activity in the western hemisphere (Wiemken et al., 2023). Data collection involved Boolean search terms (Appendix 1) to capture a wide range of discussions.

### 2.3 Developing coding and thematic guidance

The team collaboratively identified initial themes and topics during preliminary analysis discussions. The lead researcher then developed a coding framework based on these discussions, detailing specific inclusion/exclusion criteria for consistent application across the dataset. This coding guide was shared with the team for feedback and refinement, ensuring consensus, until a final inclusion and exclusion criteria was then applied (Appendix 2):

- **Inclusion:** Posts from individuals of any age with a diagnosis of Long COVID, or those receiving support for Long COVID.
- **Exclusion:** Posts unrelated to Long COVID or mental health conditions resulting from other causes. Comorbid diagnoses were included (a full explanation of these criteria is in Appendix 2).

Team members applied these criteria to identify relevant posts, enabling us to maintain a focused dataset. In line with the LISTEN process, data categorization was documented systematically using a Computer-Mediated Analysis (CMA) sheet (Vera San Juan et al., 2023), which captured key themes in social media discussions and allowed for a structured qualitative analysis of the data.

### 2.4 Data analysis (iterative digital and team analysis cycles)

This study employed a combined qualitative and digital analysis approach to ensure a nuanced and accurate interpretation of social media discourse on Long COVID. Manual qualitative analysis was a central component, conducted in parallel with digital text analysis tools. The analysis was primarily deductive, identifying themes aligned with known challenges in Long COVID. Grounded theory principles (Charmaz, 2006) were integrated into team discussions to facilitate a deeper exploration of the dataset, ensuring that themes emerged directly from the lived experiences of individuals rather than being imposed by pre-existing frameworks. Specifically, the team engaged in constant comparative analysis, iteratively reviewing and refining themes to ensure close alignment with the data. This approach allowed for nuanced patient perspectives to emerge, particularly regarding mental health and self-management practices.

While digital tools such as Infranodus were used to detect structural patterns, connections, and clusters of keywords within the 1,333 social media posts, these did not replace human analysis. Instead, digital outputs served as an initial guide, helping highlight key topics for further qualitative investigation by the research team. The dataset was manually filtered to exclude irrelevant content, ensuring that only posts directly related to Long COVID and mental health were analyzed. Importantly, qualitative coding did not rely solely on automated outputs; rather, every post was manually reviewed and coded by researchers using the LISTEN collaborative matrix approach to capture sarcasm, implicit meanings, and culturally specific expressions that might otherwise be overlooked in computational analysis.

Themes were refined through iterative analysis cycles, where emerging patterns from both manual and digital analyses were revisited and re-evaluated during collaborative team discussions. This approach ensured a balance between computational pattern detection (top-down) and human-led thematic interpretation (bottom-up), resulting in a rigorous and contextually grounded analysis.

To further ensure coding reliability, three rounds of collaborative coding meetings were conducted, during which the team reconciled discrepancies in interpretation and refined the coding framework. As a result, intercoder agreement improved from 60% to 85%, with a Fleiss' Kappa of 0.73, demonstrating substantial reliability and consistency in coding application. This systematic approach mitigated the risk of over-reliance on digital tools, reinforcing the depth and accuracy of the qualitative findings.

### 2.5 Synthesis and reporting of findings

The team consolidated findings into a coherent narrative, holding reflective discussions to address ambiguities and clarify themes. We employed data visualization tools, such as treemaps and frequency tables, to support synthesis, highlighting key themes around mental health impacts, and the varying attitudes toward supplement use. The final findings were summarized, with additional analysis conducted as needed to resolve outstanding questions. This synthesis informed our final discussion of public perceptions of Long COVID and provided a basis for recommendations on evidence-based approaches to mental health and symptom management.

### 2.6 Ethical considerations

This study collected data solely from publicly accessible social networks and implemented several measures to ensure the anonymity of individuals whose content was analyzed. Consistent with best practices in social media research (Martin et al., 2020; Ahmed et al., 2017; Ayers et al., 2018), we employed a local Python script (Kummervold et al., 2021) to anonymize all data. The script replaced usernames and links with anonymous text (e.g., @user) and summarized and paraphrased tweets to retain the original meaning while avoiding direct quotations that could be traced back to the original poster. No information that could potentially identify individuals was included in the study.

When conducting internet research, it is crucial to carefully consider whether ethics approval and informed consent are necessary (Eysenbach and Till, 2001). This project was reviewed and approved by the Humanities and Social Sciences Research Ethics Committee (HSSREC), University of Warwick (Application Reference: HSSREC 118/22-23). The Warwick study covering the analysis of Long COVID social media conversations is ongoing. Written informed consent was not obtained due to the use of anonymized secondary data derived from publicly available social media posts, in accordance with the Terms of Use of the platforms and established guidelines for secondary data research. The anonymization process adhered to the Central University Research Ethics Committee (CUREC) Ethics Guidance on Internet-mediated Research at the University of Oxford, which exempts studies using large anonymized datasets from formal ethics approval when robust pseudonymisation measures are implemented [CUREC (Central University Research Ethics Committee), 2023].

This study was conducted as part of a collaborative research effort involving the University of Warwick (data collection), University College London (secure data storage and analysis) (UCL Research Ethics, 2022), and the University of Oxford (data collection and analysis). A similar ethics approach has been used in other studies involving anonymized datasets (Déom et al., 2023). All safeguards for maintaining participant anonymity, including the use of secure encrypted servers via the Pulsar and Infranodus platforms, were strictly followed to ensure the privacy of individuals and the integrity of the research process.

The researchers adhered to the fundamental principles of ethics, including beneficence, non-maleficence, autonomy, and justice (Varkey, 2021). No direct or easily traceable quotes are included, and all usernames were anonymized prior to analysis.

## 3 Results

### 3.1 Characteristics of the dataset

**Figure 2:**
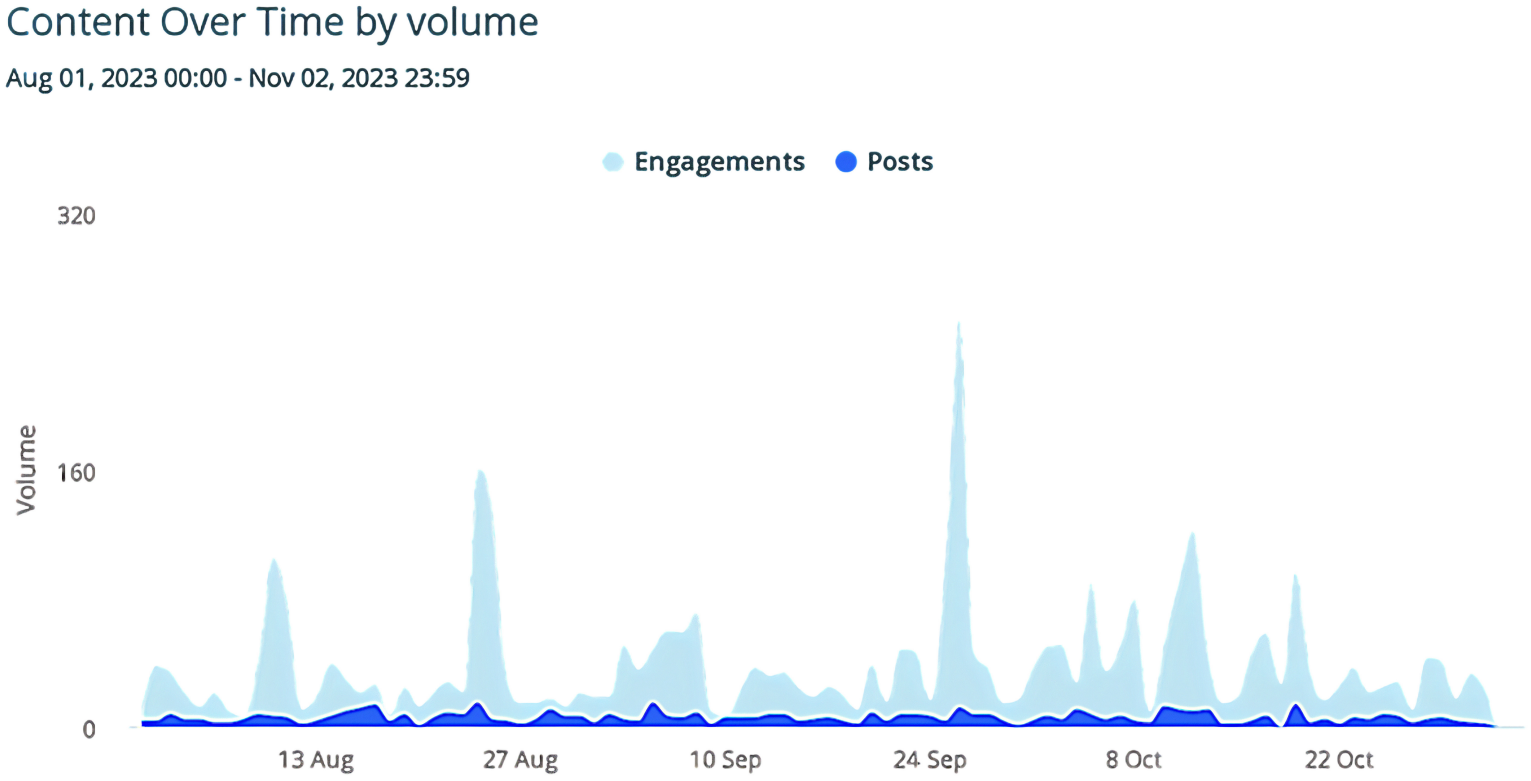
Total volume of posts over time.

Our analysis captured 5,273 pieces of content over a 93-day period (from August 1 to November 2, 2023), resulting in ~101 million potential social impressions and 43,408 engagements. This volume reflects the overall reach and level of engagement with Long COVID-related topics during this period (Figure 2).

Of the 5,273 pieces of content collected, a refined dataset was created to focus on the most relevant material. Using inclusion and exclusion criteria (Appendix 2), we analyzed 1,333 social media posts in detail. This subset included posts that directly addressed Long COVID, mental health, and supplement use, while posts unrelated to these themes were excluded. The refinement process ensured that the analysis focused on meaningful contributions to the discourse. Posts were identified across multiple platforms, including X (formerly Twitter), Facebook, and blogs, with X contributing the largest share of content.

The dataset had a limited global reach, with posts primarily originating from the United States (54.1%), United Kingdom (23.3%), and Canada (8.1%). English was the dominant language (97.4%), with smaller proportions in Polish (2%) and German (0.5%). Authors predominantly fell within the 40+ age group (47.2%), and male authors represented a higher proportion across all age categories.

### 3.2 Metrics overview

To understand the distribution and impact of Long COVID-related discussions across social media, we tracked several key metrics within Pulsar (Table 1). In this study, “engagements” refer to user interactions including likes, shares, comments, retweets, and quote-posts, as defined by the Pulsar Platform. These interactions represent public visibility and surface-level traction rather than sentiment or clinical validation.

**Table 1:** Pulsar metrics used for the descriptive analysis of posts and their descriptions.

**Metric**	**Description**
Pieces of content	Individual posts, tweets, or articles resulting from searches using specific terms.
Potential social impressions	Estimated display frequency, calculated from follower counts multiplied by content posted.
Engagements	User interactions, including likes, comments, shares, and retweets.
Demographic data	Author demographics, including age group, gender, location, and language.

### 3.3 Thematic analysis of social media posts

#### 3.3.1 Long COVID and mental health themes

The thematic analysis of the 1,333 social media posts was conducted in two stages. First, a manual analysis was performed to identify recurring mental health-related themes, focusing on key patterns observed across the dataset. Following this, Infranodus, a text network analysis tool, was used to complement the manual findings by uncovering high-level patterns and relationships among keywords and topics. This dual approach enabled a robust exploration of the dataset, identifying the following themes:

- **Mental health effects:** This theme captured a range of mental health impacts attributed to Long COVID, including trauma from symptoms, quality of life reductions (e.g., anxiety, fatigue, cognitive impairments like brain fog), and effects on relationships and employment.
- **Worries about future:** Posts frequently referenced concerns about symptom relapses, symptom fluctuation, and the unpredictability of Long COVID's trajectory.
- **Guilt and loss:** This sub-theme included expressions of guilt and sadness related to the inability to perform activities as before, as well as associated feelings of hopelessness.
- **Gender and class inequalities:** Discussions also focused on disparities in mental health experiences due to economic and social factors, noting that financial strain and job insecurity often exacerbate mental health challenges.

The initial text and topical analysis with Infranodus identified a significant connection between Long COVID and mental health issues, particularly among marginalized groups. Studies highlighted an increased risk of psychological distress among young adults with Long COVID, especially those identifying as transgender or non-binary. Young adults previously diagnosed with mental health conditions had a 35% higher likelihood of serious psychological distress associated with Long COVID, while those without prior diagnoses had an 81% increased likelihood.

There was a notable negative sentiment on social media, with many questioning why authorities were not perceived to have allocated appropriate funding to address this issue. Posts reflected concerns about gender inequality in COVID-19 treatment and care for LGBTQ+ communities. The analysis also highlighted the strong theme of Myalgic Encephalomyelitis/Chronic Fatigue Syndrome (ME/CFS), possibly due to the similarity of symptoms and experiences between ME/CFS and Long COVID. Authors described their experiences with the two syndromes co-existing, as shown by the following summarized tweet:

“*Do others with #MECFS and #LongCovid face mental health challenges during #PEM, in addition to the physical symptoms? Such as depression, emptiness, or even suicidal thoughts?” (@user, 2023)*

The group collaborative matrix analysis revealed several sub-themes related to Long COVID and mental health (Table 2). These included the worsening of mental health due to Long COVID, coping strategies, and the worry and guilt associated with decreased functioning. Users frequently mentioned the interconnectedness between mental health issues and the physical symptoms of Long COVID. The analysis also emphasized the overwhelming concern about the damaging effects of Long COVID and the negative emotions expressed in the posts. Users mentioned that Long COVID differs from other chronic illnesses and cannot be managed with lifestyle changes alone.

**Table 2:** Mental health themes identified in Long COVID social media discourse.

**Theme**	**Sub-themes**
Mental health effects	Trauma from symptom impact; Quality of life reductions: e.g., anxiety, fatigue, cognitive impairments (brain fog).
Worries about future	Relapses, symptom fluctuation, unpredictability.
Guilt and loss	Feelings of guilt, sadness, hopelessness due to inability to function as before.
Gender and class inequalities	Mental health disparities related to socioeconomic factors.

The topic of worsening existing mental health conditions and onsetting new ones was prevalent in the dataset. Long COVID and CFS were found to be distinctly related to increased experiences of poor mental health and increased rates of negative side effects on both physical and mental wellbeing, while other discussions mentioned research highlighting a discovery of an underlying biological similarity between the two (Komaroff and Lipkin, 2023).

One tweet also quoted evidence from a paper on Long COVID—that “the body of evidence on long COVID suggests SARS-CoV-2 is not only a respiratory virus; it is a systemic virus that may provoke damage and clinical consequences in nearly every organ system—including mental health disorders and neurocognitive decline” (Alwan, 2021).

#### 3.3.2 Long COVID and food supplement themes

Approximately 30% of posts discussed food supplements as a method for managing Long COVID symptoms. Key themes around supplements included (Table 3):

**Table 3:** Food supplement themes and sub-themes in Long COVID social media discourse.

**Theme**	**Sub-themes**
Symptom-specific supplement use	Discussions focused on specific supplements, such as prednisolone, nicotine patches, tramadol, benzodiazepines, creatine, and amino acids, commonly used to target symptoms like fatigue and cognitive issues.
Overall health and prevention	Some posts mentioned supplements intended for general health or to prevent further Long COVID symptoms.
Medical alternatives	Users frequently debated the validity of using food supplements in place of or alongside formal medical treatments.
Misinformation and skepticism	A significant proportion of users expressed distrust toward commercialized supplements, noting a lack of substantial scientific backing for some products, while others reported adverse reactions.

While skepticism was also present, with some users questioning the validity and effectiveness of the food supplements mentioned. The analysis also revealed concerns about the commercialization of food supplements for Long COVID treatment, with some users expressing frustration over the promotion of expensive food supplements lacking substantial scientific backing. While some users report positive experiences with food supplements, such as NAC (N-acetylcysteine) and Natto (Nattokinase) and Serra (Serrapeptase), zinc, and Vitamin D food supplements, and recommended within Long COVID support groups for managing various symptoms—many also expressed disappointments, and hopelessness about the ineffectiveness of some of the food supplements they had taken. Some users cited life-threatening incidents associated with their use, ranging from no-effect outcomes to severe adverse reactions, including disability and symptoms resembling brain embolisms. These accounts underscore the risks and uncertainties surrounding the use of food supplements as a treatment approach for Long COVID and related illnesses.

Posts also contained newly published articles relating to Long COVID. The demographic may be indirectly described as an emerging “patient-expert” community that posts, shares, and reports current and emerging research papers focused on the topic of research and management of Long COVID. These posts were often compiled with a mixture of quoted facts from papers/reports, and article links, as has been reported in other Long COVID literature (Hung et al., 2023).

**Figure 3:**
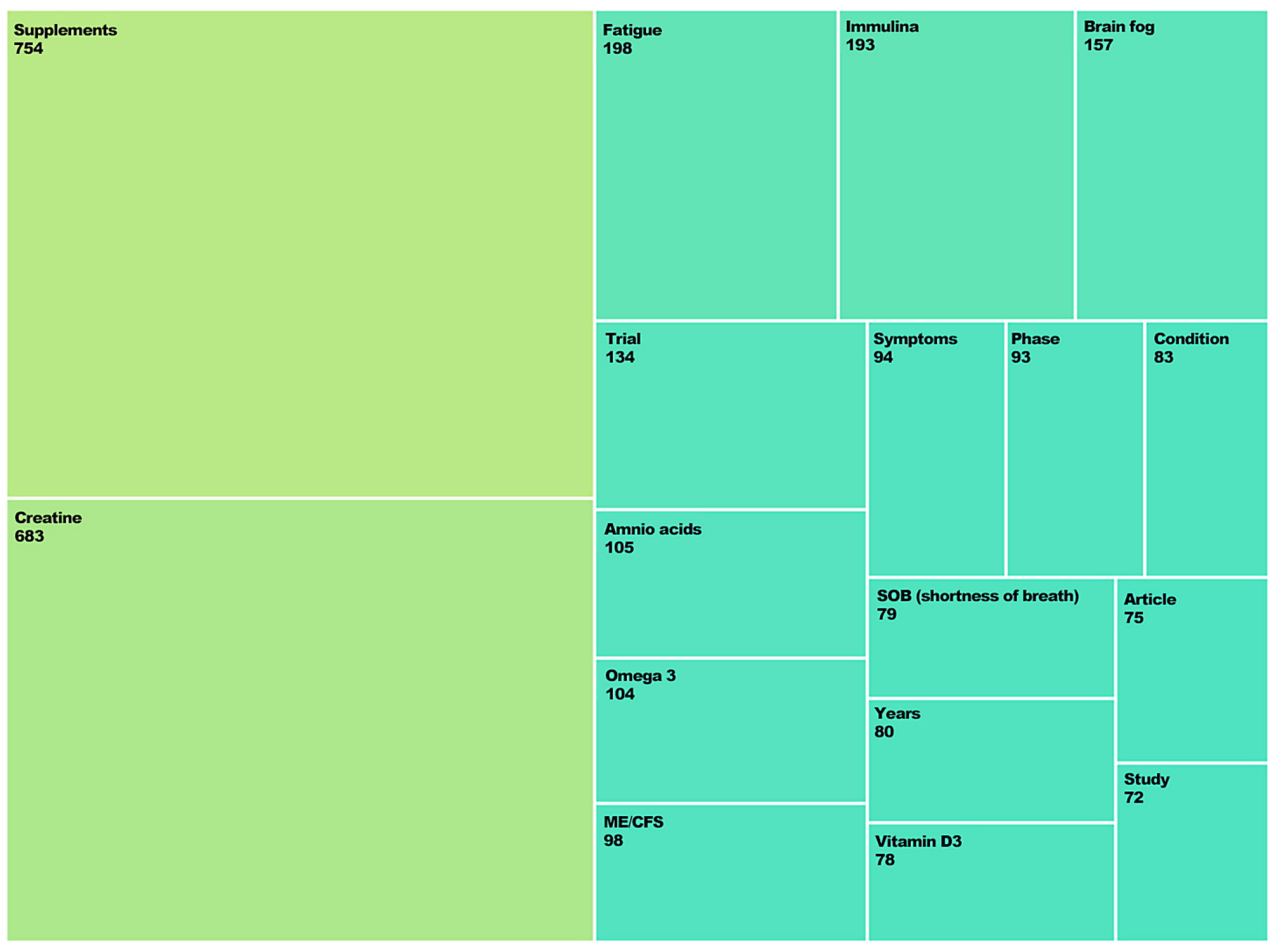
Treemap visualization of the most frequently mentioned food supplements and associated symptoms discussed in the dataset. Node size reflects volume of posts containing both terms. “Engagements” in this context refer to aggregated user interactions such as likes, retweets, shares, and comments as recorded by the Pulsar Platform.

The group collaborative matrix analysis also revealed supplement-related posts focused on prevention, symptom alleviation, specific food supplements for struggling individuals, and clinical solutions. The most mentioned food supplements included creatine, Immulina, and amino acids. These food supplements were used to target specific symptoms such as fatigue, cognitive issues, and respiratory problems. Other tweets within this theme shared personal experiences with food supplements and the reduction of problematic symptoms, encouraging others to try the treatment. The use of food supplements as symptom relief for both Long COVID and CFS was also shared and discussed (Figure 3).

Users reported both positive and negative experiences with food supplements. Some shared success stories with specific food supplements and positive access to new clinical trials, while others expressed disappointment and concerns about the ineffectiveness, high costs or potential risks associated with supplement use:

“*[…] this account is all about helping ppl with long covid/ME/CFS but they are all the time promoting super expensive stuff with little backing research kava IS a nice relaxant, but you can make your own effective tincture of it for like 1/20 the cost of these silly food supplements”*

“*Hundreds of dollars every month on food supplements and medications alone, AND I can't work. Masks are WAY cheaper than this.”*

The analysis also revealed tensions between clinical and anecdotal evidence and challenges in finding credible sources of information about food supplements for Long COVID management.

## 4 Discussion

This study employed the LISTEN method to explore the current landscape of Long COVID, focusing on its impact on mental health and the use of food supplements among affected individuals. By analyzing social media data from Pulsar and Infranodus and conducting a qualitative thematic analysis, we gained valuable insights into the experiences, perceptions, and coping strategies of Long COVID patients. This discussion will address three main areas: firstly, the mental health impacts of Long COVID, particularly in marginalized communities, and the challenges of accessing adequate support; secondly, the widespread use of food supplements as a self-directed coping strategy and the gaps in scientific evidence supporting their use; and thirdly, the emergence of patient-led initiatives, including the role of participatory research and precision medicine approaches in advancing Long COVID management.

### 4.1 Mental health impacts of Long COVID

Our analysis revealed several prominent mental health themes. For marginalized communities, including young adults and LGBTQ+ individuals, the burden of Long COVID extends beyond physical symptoms, manifesting in significant psychological distress. This aligns with findings from Vindegaard and Benros (2020) and Taquet et al. (2021), emphasizing the need for targeted mental health interventions. Social isolation, financial strain, and reduced access to reliable healthcare services emerged as recurring issues in social media discussions, reflecting how socioeconomic inequalities can exacerbate the psychological impacts of Long COVID (Taquet et al., 2021; Vindegaard and Benros, 2020).

As noted in the Introduction, the systematic review by Hawke et al. (2022) highlights the limited yet emerging research on interventions aimed at mental health, cognition, and psychological wellbeing among Long COVID patients. While some registered trials explore psychological therapies and cognitive rehabilitation to manage mental health outcomes, many of these trials are still in feasibility stages, with no established protocols specifically tailored for Long COVID patients. Our findings echo this challenge, with social media users reporting feelings of abandonment and frustration over the lack of accessible, structured mental health support.

### 4.2 Use of food supplements

The use of food supplements as a self-directed coping mechanism was another prominent theme. Social media users expressed a spectrum of attitudes, ranging from enthusiasm about emerging studies to skepticism over effectiveness and concerns about commercialization. Frequently mentioned supplements included prednisolone, nicotine patches, tramadol, benzos, creatine, and amino acids. However, as highlighted by Hawke et al. (2022) and Chandan et al. (2023), robust scientific evidence supporting the efficacy of supplements for Long COVID remains scarce (Chandan et al., 2023). Discussions on social media often centered on the financial strain caused by ongoing supplement use, particularly for those with limited disposable income, reinforcing socioeconomic barriers to symptom management.

Additionally, discussions frequently linked Long COVID supplement use to the management of other chronic illnesses, particularly ME/CFS (Myalgic Encephalomyelitis/Chronic Fatigue Syndrome). The overlap in symptoms between Long COVID and ME/CFS led to discussions about underlying biological similarities and shared experiences of symptom management, aligning with research by Davis et al. (2021) which characterized Long COVID in an international cohort (Davis et al., 2021). This connection highlights the importance of considering Long COVID within the broader context of post-viral syndromes and chronic illnesses.

However, while our study provides insights into the role of supplements in self-management, it does not seek to evaluate their clinical efficacy or establish causal relationships between supplement use and recovery outcomes. Given the lack of systematic tracking of health outcomes in social media discourse, direct causal inferences cannot be drawn from the data. Future research could address this gap by adopting longitudinal study designs, systematically tracking patient-reported health changes over time, and integrating clinical validation where feasible.

Additionally, Chandan et al.'s (2023) findings highlight the public health need for safe, scalable interventions for Long COVID (Chandan et al., 2023). While social media users often resort to supplements as a perceived solution for managing symptoms in the absence of clear clinical guidance, the risks associated with unsupervised supplement use—ranging from adverse reactions to financial burdens—show the necessity for clinically validated non-pharmacological interventions and patient education. The enthusiasm for supplements, contrasted with the limited endorsement from clinical studies, points to an urgent need for comprehensive guidelines that address both efficacy and safety, particularly in a context where patients are driven to self-manage chronic, post-viral symptoms without sufficient healthcare support.

### 4.3 Patient-led initiatives and precision medicine

A characterizing feature of the social media dataset was the emergence of a “patient-expert” community within the Long COVID discourse. These individuals actively used social media to share and discuss scientific research, personal experiences, and self-tracking data related to Long COVID management. This phenomenon reflects a broader trend of patient empowerment and participatory research in the context of emerging health issues, as described by Callard and Perego (2021) in their analysis of how patients shaped the Long COVID narrative (Callard and Perego, 2021).

Building on this, Lewthwaite et al. (2023) propose a “Treatable Traits” (TT) approach—a precision medicine framework designed specifically to address the complexity and heterogeneity of chronic conditions like Long COVID. This approach categorizes Long COVID into clusters of “treatable traits,” each linked to recognizable symptoms that can be clinically targeted. By systematically identifying these treatable traits in individual patients, such as fatigue, cognitive impairment, or respiratory limitations, this model aims to customize interventions to each patient's unique symptom profile, moving beyond the limitations of a “one-size-fits-all” approach.

In the context of the patient-expert community observed in social media posts, this approach resonates strongly. Social media users often discussed self-tracking and personalized strategies in the absence of clear treatment pathways, mirroring the TT approach's emphasis on individualized care. As Lewthwaite et al. (2023) note, establishing standardized criteria for such traits could support more structured treatment options, aligning with the grassroots movement among patients for more nuanced, adaptable Long COVID management. Together, these insights suggest that a personalized medicine model may represent a feasible path forward, both clinically and in response to the growing demand for patient-led, research-informed approaches to complex, chronic conditions like Long COVID.

### 4.4 Broader implications

Our study also highlighted the complex interplay between Long COVID, mental health, and socioeconomic factors. In particular, the disproportionate impact on individuals from marginalized communities suggests a need for public health policies that prioritize equitable access to care and tailored support systems. Social media discussions frequently revealed a lack of awareness among healthcare providers about the unique challenges faced by these populations. Discussions about occupational health, financial strain, and access to healthcare highlight the multifaceted nature of the Long COVID experience and the need for comprehensive support systems, aligning with Cutler's (2022) analysis of the economic costs of Long COVID (Cutler, 2022).

Translating these findings into real-world clinical practice, several actionable recommendations emerge. First, the prominence of mental health challenges in our dataset underscores the need for integrated care models that simultaneously address physical symptoms, psychological distress, and socioeconomic barriers. Healthcare systems could benefit from implementing standardized mental health screening for all Long COVID patients, followed by tailored psychological support integrated with symptom management. Second, given the widespread use of supplements without professional guidance, clinicians should proactively discuss self-management strategies with patients, offering evidence-based information on supplement safety and efficacy rather than leaving patients to navigate this landscape alone. Third, the emergence of patient-expert communities suggests that healthcare providers should consider establishing collaborative care frameworks that legitimize and incorporate patient expertise, potentially through peer support programmes or participatory clinical decision-making. Finally, our findings highlight the need for medical education that prepares clinicians to address the complexity of Long COVID through interdisciplinary knowledge and shared decision-making skills. By embracing these approaches, clinical practice can evolve to better address both the biomedical and psychosocial dimensions of Long COVID, reducing the current gap between patient needs and healthcare provision.

Together, these findings underscore the necessity for a multifaceted approach to Long COVID, integrating clinical, social, and psychological support within patient-centered frameworks. As social media platforms continue to serve as vital spaces for patient community building and information exchange, they also highlight the urgent need for formalized, accessible, and safe management strategies. Embracing a precision medicine framework, alongside patient empowerment, could thus play a critical role in advancing both clinical practices and public health responses to Long COVID's diverse and evolving challenges.

## 5 Limitations and future directions

While this study provides valuable insights into the public discourse on Long COVID, several limitations must be acknowledged. Social media data is inherently self-selected, meaning that individuals who do not engage in online discussions or prefer traditional healthcare channels are underrepresented. As a result, the perspectives captured may not fully reflect the experiences of all Long COVID patients, particularly those with limited internet access or engagement in patient communities.

This study utilized the LISTEN method's collaborative matrix approach, which integrated qualitative human-led analysis with digital tools. To mitigate concerns about over-reliance on digital tools, our analysis was not solely dependent on digital tools like Infranodus. Rather, three rounds of collaborative coding meetings were conducted to refine thematic interpretations, reconcile discrepancies, and ensure intercoder reliability. However, as with any qualitative approach, a degree of subjectivity remains in coding and theme development. Future research may benefit from further expanding intercoder reliability assessments or incorporating comparative analyses with alternative qualitative coding frameworks to further enhance methodological transparency. While the study does not include formal statistical modeling, we incorporated intercoder reliability metrics using Fleiss' Kappa (κ = 0.73) to ensure consistency in qualitative coding. Given the participatory and exploratory aims of the LISTEN method, we believe this provides sufficient methodological rigor within the project's qualitative scope.

Additionally, while our findings highlight the widespread use of dietary supplements among Long COVID patients, this study did not systematically measure clinical outcomes or establish causal links. Future research could adopt longitudinal designs to track patient-reported improvements and assess the efficacy of supplements in a controlled manner.

The dataset primarily consisted of English-language posts from the UK, US, and Canada, limiting the study's ability to analyze regional variations in attitudes toward supplement use and mental health. Cultural, social, and healthcare system differences may shape how Long COVID patients perceive and manage their condition, and future studies could expand to multilingual datasets for a broader perspective. Additionally, while social media provides valuable patient-centered insights, integrating clinical records or survey-based health outcomes would strengthen the reliability of findings. Ethical and privacy concerns currently restrict access to medical data, but collaborations with healthcare institutions could enable future studies to bridge this gap and validate patient-reported experiences. Additionally, while further statistical evaluation may add value in future research, the scope and funding of this project were oriented toward qualitative discourse analysis and thematic interpretation of lived experience narratives.

Despite these limitations, this study demonstrates the potential of real-world social media data in capturing patient experiences, informing public health strategies, and identifying emerging trends in Long COVID management. The findings highlight the need for further interdisciplinary research that combines digital methodologies with clinical and qualitative approaches to provide a more comprehensive understanding of Long COVID and its management.

## 6 Conclusion

This study demonstrates the effectiveness of the LISTEN method as a robust approach for analyzing large-scale social media datasets and online discussions on Long COVID, mental health, and supplement use. By integrating digital analytics tools such as Pulsar and Infranodus with collaborative human insight, we captured a nuanced understanding of the lived experiences and coping strategies of those affected by Long COVID. This aligns with the patient-centered approach advocated by Ali Awan et al. (2021) in their work on citizen scientists in Long COVID research, emphasizing the value of participatory and inclusive methodologies (Ziegler et al., 2022).

The findings of this study contribute to a better understanding of the complex nature of Long COVID and provide insights to inform the development of comprehensive, patient-centered care strategies that address both the physical and mental health needs of this population.

Our findings point to several critical areas for future research and intervention. First, there is a pressing need for targeted mental health support, particularly for vulnerable groups within the Long COVID community. As shown by Taquet et al. (2021), the incidence of mental health conditions in Long COVID highlights the urgent need for tailored interventions (Taquet et al., 2021). Additionally, exploring the biological and symptomatic overlaps between Long COVID and other post-viral syndromes, such as ME/CFS, is essential to deepen our understanding of shared mechanisms and inform more precise and effective treatment (Komaroff and Lipkin, 2023).

The study also underscores the necessity of rigorous, evidence-based research into the efficacy and safety of food supplements in Long COVID management (Lordan et al., 2021). The widespread reliance on self-managed symptom relief through supplements highlights the importance of providing patients with accessible, science-based information on safe and effective treatment options. Additionally, the emergence of a “patient-expert” community underscores the potential for patient-partnered research models, which support more inclusive and adaptable healthcare responses (McCorkell et al., 2021).

Moreover, the study underscores the need for comprehensive public health strategies that address the wider social and economic impacts of Long COVID, including school safety and occupational health protections, as highlighted by Ballering et al. (2022).

In summary, interdisciplinary, technology-enabled research approaches like LISTEN offer significant potential for addressing emerging health challenges. While our data interpretation was qualitative, future research could extend these findings using statistical modeling or hypothesis testing to quantify associations across a wider population. By leveraging social media as a tool to amplify diverse patient voices and bridging big data analysis with human insight, we can develop more effective, patient-centered strategies for Long COVID and similar complex health conditions, fostering a collaborative approach to advancing public health research.

## Data Availability

The datasets presented in this article are not readily available because the dataset contains identifiable information of users of social media posts studied for this research. The university ethics board prohibits such data to be shared or be publicly accessible. Requests to access the datasets should be directed to the corresponding author.

## Footnotes

^1^Pilates, resistance exercises, telerehabilitation, and neuromodulation are examples of non-pharmacological interventions. *Pilates* is a form of low-impact exercise that focuses on core strength, flexibility, and posture. *Resistance exercises* involve activities that improve muscle strength by working against a force, such as weights or resistance bands. *Telerehabilitation* refers to the delivery of rehabilitation services through digital platforms, allowing remote access to therapy and monitoring. *Neuromodulation* encompasses techniques such as transcranial magnetic stimulation or electrical stimulation to influence nerve activity and alleviate symptoms. *Dyspnoea* is the clinical term for shortness of breath or difficulty breathing, commonly reported in conditions affecting respiratory health.

## Appendix

### Appendix 1

#### Boolean search terms

**Main terms:** (Long covid, food supplements and mental health combined).

(“long covid” OR “longcovid”) AND (“food supplements” OR “supplement” OR “mental health” OR “mental health study” OR “mental health studies” OR “mental health research”).

**Sub-boolean search terms** (food supplements within long COVID dataset, excluding mentions of Donald Trump and the moon [presents of bots within the dataset]):

supplement*—Trump–moon.

### Appendix 2

#### Inclusion and exclusion criteria for all social media data

- Inclusion: Posts from individuals of any age with a diagnosis of Long COVID, or those receiving support for Long COVID.
- Exclusion: Posts unrelated to Long COVID or mental health conditions resulting from other causes. Comorbid diagnoses were included.

**Inclusion criteria**

1. **Individuals diagnosed with Long COVID**

Posts explicitly mentioning individuals diagnosed with Long COVID were included. These posts often referred to formal medical diagnoses or self-reported confirmations of Long COVID, with references to symptoms like fatigue, brain fog, or breathlessness persisting beyond the acute phase of COVID-19.

2. **Support for Long COVID** Posts from individuals receiving support for Long COVID, such as participation in online support groups, community forums, or advocacy groups, were included. Support could involve emotional, medical, or practical assistance aimed at managing Long COVID symptoms.

3. **Symptom descriptions consistent with Long COVID** Posts describing symptoms commonly associated with Long COVID (e.g., chronic fatigue, dyspnoea, musculoskeletal pain, cognitive impairments) were included even if no formal diagnosis was mentioned, provided the context clearly indicated the individual's struggle with post-viral conditions attributable to COVID-19.

**Exclusion criteria**

1. **Unrelated to Long COVID**

Posts that did not mention Long COVID or discuss its symptoms, treatment, or impact were excluded. General discussions about COVID-19, vaccinations, or public health measures without reference to Long COVID were excluded to maintain focus.

2. **Mental health conditions from non-COVID Causes**

Posts addressing mental health conditions unrelated to Long COVID were excluded. For example, posts solely discussing pre-existing conditions, such as generalized anxiety or unrelated mental health disorders, without reference to Long COVID, were not considered.

3. **Posts not reflective of personal or community experience**

Posts that were purely promotional (e.g., advertisements for supplements or treatments) or generated by bots were excluded. Similarly, purely informational posts without evidence of personal or community engagement, such as news articles or medical advice with no user commentary, were excluded.

4. **Comorbid diagnoses**

While comorbidities (e.g., Long COVID with pre-existing conditions like asthma or diabetes) were included, posts exclusively focused on these other conditions without mention of Long COVID were excluded.
